# Safety and operational feasibility of single versus cocktail rabies monoclonal antibodies for post-exposure prophylaxis in a resource-limited setting

**DOI:** 10.1371/journal.pntd.0014274

**Published:** 2026-07-23

**Authors:** Nidhi Fotedar, Haradanahalli Shankariah Ravish

**Affiliations:** 1 Formerly with the Department of Community Medicine, Kempegowda Institute of Medical Sciences, Hospital & Research Centre, Bengaluru, India; 2 Department of Community Medicine, Kempegowda Institute of Medical Sciences, Hospital & Research Centre, Bengaluru, India; University of Calgary, CANADA

## Abstract

**Background:**

Rabies is almost universally fatal once symptoms develop but is preventable through timely post-exposure prophylaxis (PEP). Global shortages of human and equine rabies immunoglobulin (RIG) have led the World Health Organization to recommend human monoclonal antibodies (mAbs) as acceptable alternatives. Comparative real-world data on single-antibody versus two-antibody cocktail mAbs are limited, particularly from endemic, resource-limited settings.

**Methods:**

A comparative observational study was conducted in a high-volume rabies PEP clinic in India between October 2021 and December 2022. Consecutive patients with category III animal exposures received either a single human anti-rabies mAb (Rabishield; n = 159) or a two-mAb cocktail (Twinrab; n = 159) in combination with WHO-recommended vaccine. Primary outcomes included safety, ease of administration, and operational feasibility. Data were analysed using independent t-tests and chi-square or Fisher’s exact tests, with p < 0.05 considered significant.

**Results:**

Mean age was 29.7 ± 19.4 years in the Rabishield group and 32.8 ± 20.4 years in the Twinrab group. Ease of infiltration was rated “easy” in 66.7% versus 62.9% of patients (p = 0.63). Local adverse events were uncommon, with pain reported in 2.5% versus 7.5% (p = 0.07) and erythema in ≤ 1.3% of participants. No serious systemic adverse events occurred. Rabishield required more vials per patient (2.28 ± 0.84 vs 2.00 ± 0.69; p = 0.001) and had a higher mean infiltration volume (4.7 ± 1.9 mL vs 4.0 ± 1.6 mL; p < 0.001), while wastage was similar between groups (1.03 ± 0.72 mL vs 1.03 ± 0.69 mL; p = 0.95).

**Conclusions:**

Both single and cocktail human anti-rabies mAbs were safe, well tolerated, and operationally feasible in a programmatic setting. The slightly higher volume and vial requirement for Rabishield may have cost and logistics implications. These findings support broader mAb adoption to address RIG shortages in rabies-endemic, resource-limited settings.

## Author summary

Rabies is a deadly but preventable disease that continues to cause thousands of deaths each year, mostly in resource-limited settings. After a high-risk animal bite, prompt post-exposure prophylaxis (PEP) with wound washing, vaccination, and injection of rabies-specific antibodies can prevent the disease. The antibodies are usually given as rabies immunoglobulin (RIG), but RIG is expensive, in short supply, and difficult to store and use in many parts of the world. The World Health Organization now recommends human monoclonal antibodies (mAbs) as a safe and effective alternative. In this study, we compared two types of mAbs: a single antibody (Rabishield) and a two-antibody cocktail (Twinrab) in a busy rabies treatment clinic in India. We found that both products were safe, well tolerated, and practical to use in real-life clinic settings. Rabishield required slightly more vials and a higher volume for injection, which may affect cost and logistics, but overall both were effective alternatives to RIG. These results can help guide rabies prevention programs in choosing antibody products when RIG is not available.

## Introduction

Rabies is a critical public health concern in India, characterized as a dangerous zoonotic disease caused by the Lyssavirus type 1. The disease leads to acute, progressive encephalomyelitis, with an alarming case-to-fatality rate nearing 100%, making it one of the deadliest infectious diseases known, despite being nearly completely preventable through timely intervention. [1,2]. Approximately 40% of rabies victims are children, typically bitten on the face or neck, underscoring the vulnerability of this demographic [2]. The disease is endemic in more than 150 countries and territories across all continents except Antarctica, with the majority of human deaths (over 95%) reported in Africa and Asia [3]. Endemic to several continents, it is estimated that around 59,000 people die each year in the world, 40% of whom are children under 15 years of age [3]. The epidemiological landscape of rabies reveals distinct patterns of transmission and risk factors. Canine rabies is primarily responsible for the vast majority of human rabies cases, with dog bites being the leading cause of exposure [4]. Studies have shown that children aged 5–14 years are particularly vulnerable, accounting for a significant proportion of reported cases [4]. The high incidence of dog bites among children can be attributed to their interactions with dogs and lower awareness of rabies risks [4].

Additionally, stray dogs are more likely to transmit rabies than owned pets, as they tend to have greater exposure to the virus [4]. Geographical and socio-economic factors also influence the incidence of rabies. In regions with higher dog populations and where stray dogs are prevalent, the risk of transmission increases significantly [5]. In contrast, areas such as Australia, Japan, and much of Western Europe have effectively controlled rabies among dogs, resulting in lower risks of human infection [3].

The estimated global cost of rabies, including lost lives and medical expenses, is around US$8.6 billion per year, highlighting the economic burden of this neglected tropical disease [3,6]. The burden of rabies disproportionately affects low-income populations, particularly in rural areas where healthcare access is limited. Families may face costs exceeding $100 for post-exposure prophylaxis (PEP) in regions where the average income is less than $1 per day [7,8]. In addition to direct financial costs, there are indirect costs associated with lost workdays and travel to healthcare facilities for multiple vaccinations, which can be particularly challenging for low-income families [7].

The administration of post-exposure prophylaxis (PEP) for rabies is critical in preventing the onset of this fatal disease. Current guidelines recommend a combination of wound care and vaccination alongside the administration of rabies immunoglobulin (RIG) and monoclonal antibodies (mAbs) for optimal effectiveness [9]. Monoclonal antibodies (mAbs) play a crucial role in the post-exposure prophylaxis (PEP) of rabies, providing an effective means of passive immunization against the virus. They are engineered to target specific antigens, allowing for a more precise response compared to traditional polyclonal antibodies.

The safety profiles of monoclonal antibodies (mAbs) used for rabies post-exposure prophylaxis have been the focus of several studies, indicating a generally satisfactory safety profile along with improved tolerability. One significant mAb developed for rabies PEP is docaravimab/miromavimab, commercially known as Twinrab, which received orphan status from the FDA and was approved in 2019. A phase 2/3 trial in India demonstrated that a single dose of 40 IU/kg Twinrab was non-inferior in safety and efficacy to the standard 20 IU/kg Human Rabies Immune Globulin (HRIG) in patients exposed to rabies virus [10–12]. Additionally, the Serum Institute of India launched Rabishield, the first monoclonal antibody formulation for rabies, aimed at significantly reducing mortality rates associated with the disease in India [13].

Clinical trials have revealed that mAbs can be administered safely in high-risk patients, including pregnant women, children, and individuals with compromised immune systems, highlighting their potential advantage over conventional therapies [2,9,14].

However, while efficacy and safety of mAbs are well-documented, real-world data comparing operational feasibility, ease of administration, and resource utilization between single-agent and cocktail mAbs remain scarce. This study evaluates these parameters in a pragmatic clinical setting in India, directly comparing Rabishield and Twinrab in patients with category III exposures. We hypothesized that single and cocktail rabies monoclonal antibodies would demonstrate similar safety and operational feasibility profiles when administered as part of routine post-exposure prophylaxis for Category III exposures in a high-volume, resource-limited rabies clinic.

## Methods

### Ethics statement

This study was approved by the Institutional Ethics Committee of KIMS Hospital (Ref. KIMS/IEC/D-06/2021).Informed consent was obtained from all participants or their legal guardians before their inclusion in the study. For participants aged 2–17 years, written informed consent was obtained from their parents or legal representatives, and written informed assent was obtained from participants aged 7–17 years. The consent process included a detailed explanation of the study’s purpose, procedures, potential risks, and benefits.

### Study design and setting

This was a pragmatic, observational, comparative study conducted at the Anti-Rabies Clinic of Kempegowda Institute of Medical Sciences (KIMS) Hospital and Research Centre, Bangalore, India, a tertiary-care referral centre, from October 2021 to December 2022. The study compared operational parameters of a single monoclonal antibody (Rabishield, Serum Institute of India) versus a monoclonal antibody cocktail (Twinrab, Zydus Cadila) in patients with WHO category III rabies exposures. The study complied with the Declaration of Helsinki and the International Council for Harmonisation (ICH) Good Clinical Practice guidelines. Ethics approval was obtained from the Institutional Ethics Committee of KIMS Hospital (Ref. KIMS/IEC/D-06/2021). Written informed consent was obtained from all participants or legal guardians; for participants aged 7–17 years, written assent was also obtained.

### Participants

Between October 2021 and December 2022, 912 animal bite victims presented for rabies post-exposure prophylaxis (PEP). Eligibility required WHO category III exposure, presentation within seven days, and ability to provide informed consent; individuals with prior pre-exposure or post-exposure prophylaxis, PEP initiated elsewhere, unavailability for follow-up, or residence too distant for scheduled visits were excluded, yielding 758 eligible victims. Those who refused consent or were aged under 18 years without an accompanying parent or guardian were excluded, leaving 735 participants. Recipients of equine or human rabies immunoglobulin (ERIG/HRIG) were then excluded; the remaining monoclonal antibody recipients formed the study population. From these, equal numbers were included for analysis: 159 received single mAb (Rabishield) and 159 received a mAb cocktail (Twinrab). Participants were enrolled consecutively without formal sample-size calculation, reflecting a pragmatic, real-world design, and were followed for adherence to PEP and adverse events.

### Post-exposure prophylaxis procedures followed in the study

All participants received PEP in accordance with National Centre for Disease Control (NCDC) guidelines. Wounds were washed immediately with soap and running water for up to 15 minutes, followed by povidone-iodine application. Vaccination followed the intramuscular Essen regimen on days 0, 3, 7, 14, and 28, using either purified Vero cell rabies vaccine (PVRV; 0.5 mL per dose) or purified chick embryo cell vaccine (PCECV; 1.0 mL per dose), as available.

**Rabishield(single mAb):** 3.33 IU/kg (40 IU/mL; 100 IU/2.5 mL vial); **Dose (mL) = (3.33 × body weight [kg]) / 40** (i.e., 0.0833 mL/kg).

**Twinrab(mAb cocktail):** total 40 IU/kg (100 IU/mL; 400 IU/4 mL vial); **Dose (mL) = (40 × body weight [kg]) / 100** (i.e., 0.4 mL/kg).

### Mode of administration of single and cocktail monoclonal antibodies

For both Rabishield and Twinrab, the calculated dose was infiltrated directly into and around all category III wounds to maximise local neutralisation. The proportion infiltrated locally was determined by wound size and feasibility; any remaining volume was administered deep intramuscularly at a site distant from the vaccine injection site. In patients with multiple wounds, the dose was apportioned across wounds, with dilution as needed to ensure complete coverage. All administrations followed NCDC guidance with aseptic technique and avoidance of pooling beyond wound margins.

### Outcome measures

The primary outcomes were the mean number of vials used per patient and mean drug wastage per patient (mL). Secondary outcomes included the incidence of local adverse events (pain, erythema, tenderness, induration) within 48 hours and at days 3, 7, and 28, and systemic adverse events (fever, malaise, headache) over the same period. Adverse events were evaluated for causality and graded as mild (Grade 1), moderate (Grade 2), or severe (Grade 3) according to impact on daily activities. Operational feasibility parameters included ease of infiltration (easy, moderate, difficult), need for dilution, number of injection sites, time required for administration (minutes), and mAb infiltration volume (mL). Baseline characteristics (age, sex, exposure type and site, and number of wounds) were recorded at enrolment to compare groups. All variables were documented on a standardised case record form at baseline and predefined follow-up visits.

### Data collection

Data were captured on a predesigned case record form. Baseline variables included age, sex, exposure category, wound site, and number of wounds. During PEP administration, total calculated dose, number of vials opened, mAb infiltration volume, and unused residual volume were recorded. Wastage (mL) was calculated as the total volume from opened vials minus the volume administered. Adverse events were documented during immediate post-administration observation and at follow-up, with onset, duration, severity grade, and suspected causality. Events were managed according to institutional protocols and were reported through the institutional adverse event reporting mechanism, and where applicable, were notified through the national Adverse Events Following Immunization (AEFI) reporting pathway.

### Statistical analysis

Descriptive statistics are reported as mean (SD) for approximately normally distributed continuous variables and as frequency (percentage) for categorical variables; where distributions were skewed (e.g., age), medians and interquartile ranges are also presented. The two co-primary outcomes, number of vials used per patient and drug wastage per patient (mL) were compared between groups using two-sample *t*-tests. Other continuous measures (infiltration volume, number of injection sites, time to administer) were analysed similarly. Categorical variables (ease of infiltration, need for dilution, local and systemic adverse events) were compared using the Chi-square test or Fisher’s exact test, as appropriate.. All tests were two-sided with α = 0.05. Statistical analysis was performed using R software Version 2023.12.1 + 402 and the R Commander statistical packages. In addition to p-values, 95% confidence intervals were calculated for key mean differences and risk differences and are presented in the Figs.

## Results

### Participant disposition

**Fig 1:**
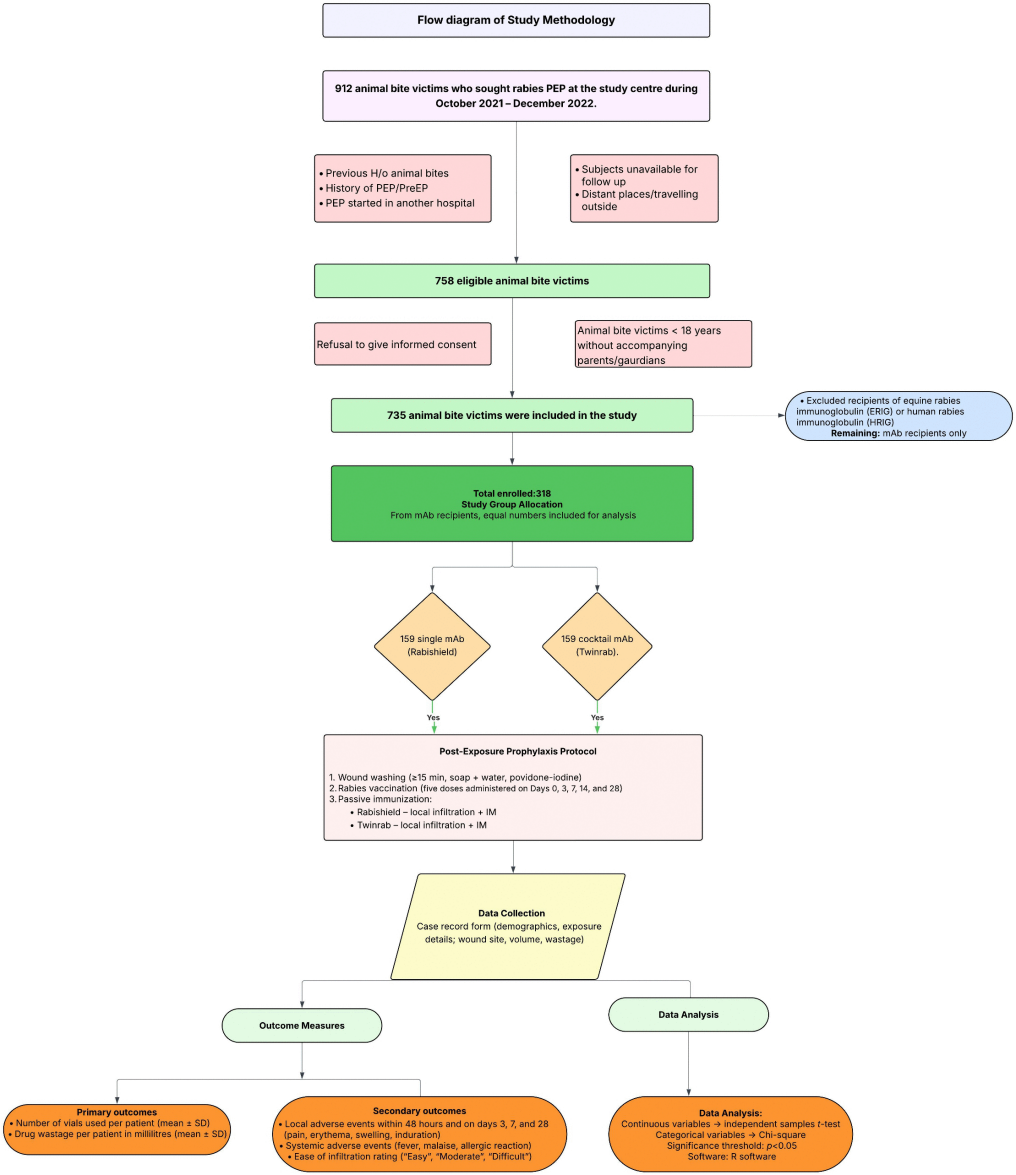
Flow diagram of participant selection and study group allocation. Of 912 animal bite victims presenting for rabies post-exposure prophylaxis (PEP) during October 2021–December 2022, 758 met eligibility criteria. Among these, 23 refused consent and 22 were excluded because they were aged <18 years without a parent/guardian present, leaving 735 participants. Recipients of equine or human rabies immunoglobulin (ERIG/HRIG) were excluded. The final analytical sample included 318 monoclonal antibody recipients, comprising 159 who received single monoclonal antibody (Rabishield) and 159 who received monoclonal antibody cocktail (Twinrab).

Of 912 animal bite victims presenting for rabies post-exposure prophylaxis between October 2021 and December 2022, 318 were included in the analysis: 159 received single monoclonal antibody (Rabishield) and 159 received a monoclonal antibody cocktail (Twinrab). The selection pathway is shown in Fig 1.

### Baseline characteristics

**Fig 2:**
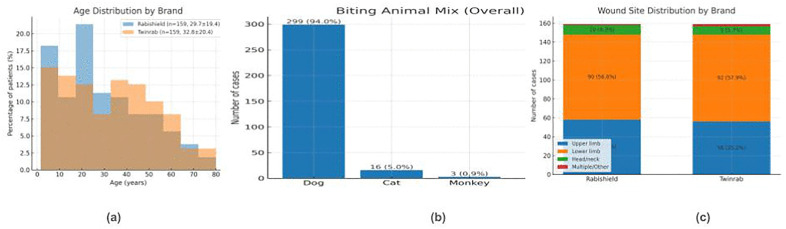
Baseline distributions by product. **(a)** Age distribution for Rabishield and Twinrab (n = 159 per group); mean age 29.7 (SD 19.4) and 32.8 (20.4) years, respectively. **(b)** Biting animals overall: dog 299/318 (94.0%), cat 16/318 (5.0%), monkey 3/318 (0.9%). **(c)** Wound-site distribution by product—Rabishield vs Twinrab: upper limb 57/159 (35.8%) vs 53/159 (33.3%); lower limb 90/159 (56.6%) vs 90/159 (56.6%); head/neck 9/159 (5.7%) vs 8/159 (5.0%); multiple/other 3/159 (1.9%) vs 8/159 (5.0%); groups comparable (χ² *p* = 0.60). See Table 1 for summary statistics.

The two groups were broadly similar at enrolment (Table 1). Mean age was 29.7 (SD 19.4) years in the Rabishield group and 32.8 (20.4) years in the Twinrab group (*p* = 0.17); the distribution of age categories did not differ (*p* = 0.21). Sex distribution (male 61.0% vs 58.5%; *p* = 0.73), provoked versus unprovoked exposure (both 34.0% vs 66.0%; *p* > 0.99), number of wounds (2.1 [1.3] vs 2.2 [1.2]; *p* = 0.70), and wound-site distribution (*p* = 0.60) were comparable. The required infiltration volume at treatment was higher with Rabishield than with Twinrab (4.7 [1.9] mL vs 4.0 [1.6] mL; *p* < 0.001). Baseline distributions are shown in Fig 2a–2c) (age distribution, biting-animal mix, and wound-site distribution)..

**Table 1:** Baseline characteristics of participants.

Characteristic	Rabishield (n = 159)	Twinrab (n = 159)	p value
Age, mean ± SD (years)	29.7 ± 19.4	32.8 ± 20.4	0.17
Median (IQR)	25.0 (13.5–42.5)	33.0 (14.5–47.0)	–
Age group, n (%)			0.21
• 0–12 years	38 (23.9%)	36 (22.6%)	
• 13–17 years	8 (5.0%)	10 (6.3%)	
• 18–30 years	49 (30.8%)	32 (20.1%)	
• 31–50 years	37 (23.3%)	45 (28.3%)	
• > 50 years	27 (17.0%)	36 (22.6%)	
Sex, n (%)			0.73
• Male	97 (61.0%)	93 (58.5%)	
• Female	62 (39.0%)	66 (41.5%)	–
Type of exposure, n (%)			>0.99
• Provoked	54 (34.0%)	54 (34.0%)	–
• Unprovoked	105 (66.0%)	105 (66.0%)	–
Number of wounds, mean ± SD	2.1 ± 1.3	2.2 ± 1.2	0.70
Site of wound, n (%)			0.60
• Upper limb	57 (35.8%)	53 (33.3%)	
• Lower limb	90 (56.6%)	90 (56.6%)	
• Head/neck	9 (5.7%)	8 (5.0%)	
• Multiple/ Other sites*	3 (1.9%)	8 (5.0%)	
RIG infiltration volume, mean ± SD (mL)	4.7 ± 1.9	4.0 ± 1.6	<0.001*

*Multiple/other sites include mixed locations such as trunk with limb wounds.

*p < 0.05 considered statistically significant.

### Primary outcomes

**Fig 3:**
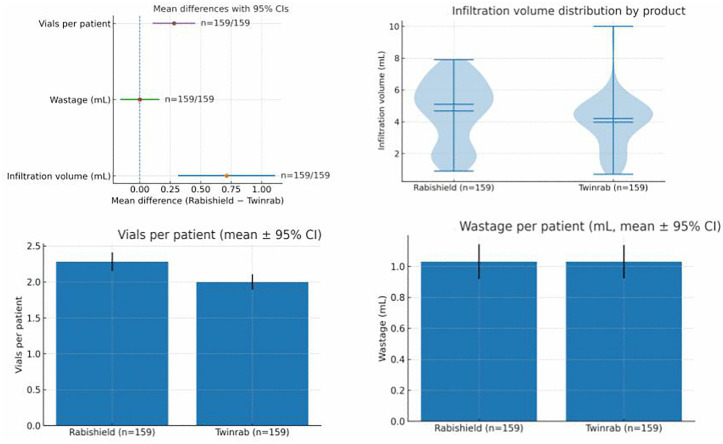
Primary and key operational outcomes comparing Rabishield and Twinrab (n = 159 per group). The upper-left panel shows mean differences with 95% confidence intervals for infiltration volume, vials per patient, and wastage per patient, with positive values indicating higher values for Rabishield. The upper-right panel shows the distribution of infiltration volume by product (violin plots). The lower-left panel shows the mean number of vials used per patient with 95% confidence intervals. The lower-right panel shows the mean wastage per patient (mL) with 95% confidence intervals. Vials per patient and wastage data were derived from Table 4, while infiltration volume was derived from individual-level data.

Rabishield required more vials per patient than Twinrab (2.28 [0.84] vs 2.00 [0.69]; *p* = 0.001) (Table 4). Mean drug wastage per patient was identical between groups (both 1.03 mL; *p* = 0.95). Primary outcome contrasts are illustrated in Fig 3.

**Table 4:** Tolerability and operational feasibility.

Parameter	Rabishield (n = 159)	Twinrab (n = 159)	p value
Ease of infiltration, n (%)			
• Easy	106 (66.7%)	100 (62.9%)	0.63
• Moderate	30 (18.9%)	37 (23.3%)	
• Difficult	23 (14.5%)	22 (13.8%)	
Need for dilution, n (%)			
Yes	20 (12.6%)	32 (20.1%)	0.09
No	139 (87.4%)	127 (79.9%)	
Number of injection sites, mean ± SD	2.18 ± 1.18	2.20 ± 1.08	0.88
Time to administer (minutes), mean ± SD	4.18 ± 1.18	4.20 ± 1.08	0.88
Vials used per patient, mean ± SD	2.28 ± 0.84	2.00 ± 0.69	0.001*
Wastage (mL), mean ± SD	1.03 ± 0.72 mL	1.03 ± 0.69 mL	0.95

*p-value calculated using independent samples t-test; p < 0.05 considered statistically significant.

### Operational feasibility

**Fig 4:**
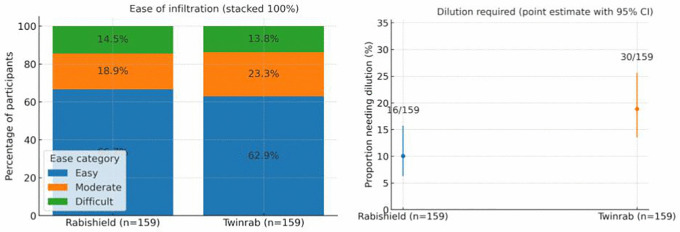
Operational feasibility profiles by product (n = 159 per group). The left panel shows the distribution of ease of infiltration as stacked 100% bar charts for Rabishield and Twinrab, with proportions corresponding to those presented in Table 4. The right panel shows the proportion of participants requiring dilution with 95% Wilson confidence intervals; counts are displayed above each estimate.

**Fig 5:**
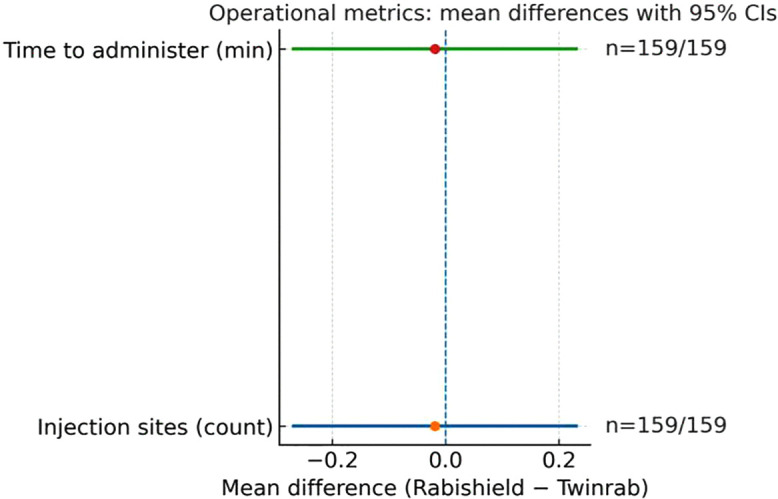
Mean differences (Rabishield − Twinrab) with 95% confidence intervals for operational metrics (n = 159 per group). The forest plot compares time to administer (minutes) and the number of injection sites between treatment groups. Values close to zero indicate no meaningful difference between Rabishield and Twinrab.

**Fig 6:**
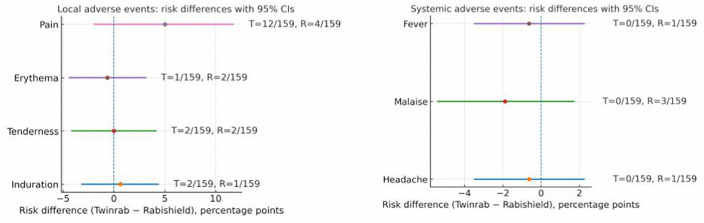
Safety outcomes expressed as risk differences (Twinrab − Rabishield) with 95% confidence intervals (n = 159 per group). The left panel presents local adverse events (pain, erythema, tenderness, and induration), while the right panel presents systemic adverse events (fever, malaise, and headache). Labels indicate the number of participants in each group (T = Twinrab; R = Rabishield). Risk differences are expressed in percentage points. Newcombe–Wilson confidence intervals were used to improve interval estimation for infrequent events.

**Fig 7:**
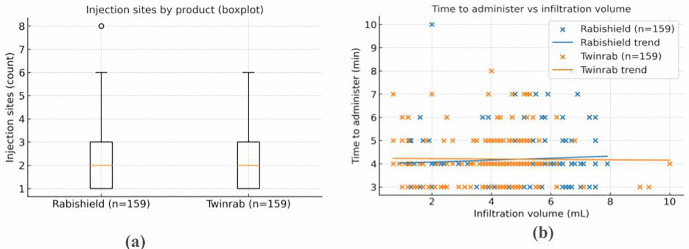
Additional operational outcome visualizations by product (n = 159 per group). The left panel shows the distribution of the number of injection sites per patient for Rabishield and Twinrab using boxplots. Boxes represent the interquartile range (IQR), the centre line indicates the median, whiskers represent the range, and points indicate outliers. Mean (SD) values were 2.18 (1.18) for Rabishield and 2.20 (1.08) for Twinrab, with a mean difference of −0.02 (95% CI −0.27 to 0.23; p = 0.88, two-sample t-test). The right panel shows the relationship between administration time (minutes) and infiltration volume (mL) for Rabishield and Twinrab. Lines represent ordinary least-squares regression fits (Rabishield slope ≈ +0.04 min/mL; Twinrab slope ≈ −0.01 min/mL), demonstrating minimal association between infiltration volume and administration time.

Ease of infiltration did not differ (easy in 66.7% vs 62.9%; *p* = 0.63). The need for dilution was more frequent with Twinrab (20.1% vs 12.6%), although this difference was not significant (*p* = 0.09). The number of injection sites (2.18 [1.18] vs 2.20 [1.08]; *p* = 0.88) and time to administer (4.18 [1.18] vs 4.20 [1.08] minutes; *p* = 0.88) were similar. Operational profiles are summarised in Figs 4 and 5. Key operational and safety outcomes are additionally presented with 95% confidence intervals in Figs 3, 5, and 6. Additional visualizations of injection-site distribution and administration time are shown in Fig 7.

### Safety

Local reactions were uncommon: pain occurred in 2.5% with Rabishield and 7.5% with Twinrab (*p* = 0.07); erythema (1.3% vs 0.6%), tenderness (1.3% vs 1.3%), and induration (0.6% vs 1.3%) were rare, with no significant between-group differences (all *p* > 0.99) (Table 2). Systemic adverse events were infrequent in both groups (fever 0.6% vs 0%; malaise 1.9% vs 0%; headache 0.6% vs 0%), with no significant differences (Table 3). Risk-difference plots for local and systemic adverse events are shown in Fig 6.

**Table 2:** Local adverse events.

Local reaction	Rabishield (n = 159)	Twinrab (n = 159)	p value
Pain	4 (2.5%)	12 (7.5%)	0.07
Erythema	2 (1.3%)	1 (0.6%)	>0.99
Tenderness	2 (1.3%)	2 (1.3%)	>0.99
Induration	1 (0.6%)	2 (1.3%)	>0.99

**Table 3:** Systemic adverse events.

Systemic reaction	Rabishield (n = (n = 159)	Twinrab (n = 159)	p value
Fever	1 (0.6%)	0	>0.99
Malaise	3 (1.9%)	0	0.25
Headache	1 (0.6%)	0	>0.99

**Missing data:** Adverse event symptom documentation was incomplete for a subset of participants. Pain, tenderness, swelling/induration, and erythema were not recorded for 155 participants; malaise was not recorded for 315 participants; and fever and headache were not recorded for 317 participants. All other study variables were complete. Analyses of individual symptoms were based on available documented data.

## Discussion

This study provides operational evidence that both the single human monoclonal antibody (Rabishield) and the two-antibody cocktail (Twinrab) can be integrated into routine rabies post-exposure prophylaxis (PEP) services without major differences in administration workload, wastage, or safety outcomes. The two groups were demographically and clinically similar, reducing the likelihood of confounding. Age and sex distribution were comparable to other Indian post-exposure prophylaxis (PEP) cohorts, where children and young adults form a substantial proportion of cases and males predominate due to greater outdoor exposure [15]. About two-thirds of exposures were unprovoked, consistent with previous hospital-based series [16], and lower limbs were the most common site, as reported in other endemic settings [16,17]. The only statistically significant baseline difference was a higher mean infiltration volume in the Rabishield group (4.7 mL vs 4.0 mL), most likely attributable to formulation concentration rather than wound characteristics. This observation mirrors findings from Bookstaver et al., who reported that variations in administered RIG volume across formulations did not affect protective efficacy when infiltration was performed meticulously into and around all wounds, as per WHO recommendations [18]. In both studies, the emphasis on wound-directed administration rather than fixed total volume appears to be the critical determinant of effective local virus neutralisation.

Notably, approximately one-quarter of participants in each group were aged ≤12 years (Table 1), supporting the feasibility of integrating both monoclonal antibody formulations into routine post-exposure prophylaxis workflows even in pediatric patients managed in similar high-volume clinic settings.

Local reactions were infrequent and mild across both products, echoing findings from a phase-3 non-inferiority trial comparing the docaravimab/miromavimab cocktail (Twinrab) with human RIG; the trial reported predominantly local, self-limited reactions and no serious safety signals [10]. Indian post-marketing surveillance of Twinrab, including the study by Shewale et al. (2020), also showed low rates of local adverse events under routine infiltration practice, and active safety monitoring of single-agent Rabishield reported similarly excellent tolerability [19–22]. The small, non-significant increase in reported pain in our Twinrab group is clinically plausible given meticulous wound-directed infiltration, which though critical for local virus neutralisation can cause transient discomfort irrespective of formulation. Although pain was numerically higher in the Twinrab group, the difference did not reach statistical significance (p = 0.07) and events were mild and self-limited, suggesting limited clinical significance.

Systemic symptoms were rare and self-limited, consistent with outcomes seen in controlled trials and real-world data. The phase-3 Twinrab trial and programmatic studies reported few systemic events, while a double-blind phase-3 study of the human monoclonal ormutivimab also demonstrated non-inferior immunogenicity to RIG and a favourable safety profile [10,19,20,23]. Mild systemic symptoms such as fever or headache are also expected following the rabies vaccine component itself and resolve without sequelae [24].

Across clinical trials, surveillance data, and practice guidelines, human or humanised anti-rabies mAbs demonstrate a robust safety profile: mostly mild local reactions, infrequent systemic symptoms, and no serious adverse events when administered via infiltration into all wounds per evidentiary guidance [10,18–24].

Across workflow measures, ease of infiltration, number of injection sites, and time to administer the two products were indistinguishable, suggesting that once standard wound-infiltration technique is applied, bedside feasibility is driven more by practice than by product identity. This aligns with prior reports describing rabies mAbs as operationally compatible with routine PEP when delivered by trained staff, and with real-world series reporting smooth integration into clinic workflows [19–22].

The need for dilution was more frequent with the cocktail (Twinrab), a finding that is clinically coherent given its higher IU/kg dosing and the need to expand volume with saline to ensure complete wound coverage. Existing guidance has long endorsed dilution “as needed” to achieve thorough infiltration, prioritising coverage over fixed undiluted volumes [12]. This pattern—more frequent dilution without added time or injection sites—has also been noted in operational evaluations [19–22].

The only statistically significant operational difference was vials per patient, modestly higher with Rabishield (2.28 vs 2.00), a reflection of presentation size and concentration. Programmatically, this means slightly more vial openings, but wastage was identical between products, implying that careful dose apportionment effectively offsets any added openings. Importantly, large-scale operational audits, such as the seven-state survey by Hanumanthaiah et al., have highlighted that wastage minimisation and steady availability depend more on procurement practices, stock monitoring, and staff training than on the inherent properties of the passive product. In that survey, facilities using careful multi-patient vial-sharing protocols and trained infiltration techniques reported low wastage despite varying formulations—mirroring our finding of parity in wastage between the two mAbs [25].

From a health-systems perspective, where the passive component is often the bottleneck (given patchy RIG and mAb supply in many Indian public facilities), the absence of differences in infiltration ease, injection-site counts, or wastage suggests that both products are operationally feasible within similar high-throughput clinic settings. However, these findings should not be interpreted as proof of universal interchangeability across all program contexts, as differences in staffing, training, patient volume, and supply-chain practices may influence feasibility outcomes. Procurement contracts, storage logistics, and local familiarity may therefore remain important determinants of product selection in real-world implementation [25].

Strengths of our study include its pragmatic, single-centre design reflecting routine practice; consecutive enrolment in a high-volume anti-rabies clinic; balanced groups receiving licensed products; and prespecified, operationally meaningful endpoints (vials per patient, wastage, infiltration workload). Data were captured on a standardised case record form with contemporaneous entry; adverse events were ascertained immediately post-administration and at scheduled visits (days 3, 7, 14, 28, and 180) with explicit severity grading. Participant flow matched the methods Fig, and analyses followed a prespecified plan with two-sided tests and 95% CIs, enhancing internal consistency and programme relevance.

Limitations of this study include its pragmatic observational, single-centre design with non-random allocation driven by product availability, which may introduce selection bias and residual confounding and precludes causal inference. Additionally, we did not include a human rabies immunoglobulin (HRIG) comparator group, limiting direct operational comparison with the conventional standard of care. However, baseline characteristics were broadly comparable between groups, suggesting minimal imbalance in measured variables; nevertheless, unmeasured confounding cannot be excluded. The sample was not powered for very rare adverse events; some operational fields (notably dilution) relied on free-text entries, introducing potential misclassification; and late adverse events partly depended on patient-held cards, with possible under-reporting. We did not collect long-term clinical effectiveness outcomes (e.g., rabies incidence or serological responses) or formal cost outcomes, and generalisability beyond similar high-throughput clinics in endemic settings should be interpreted with caution. Although the sample size was adequate for operational comparisons, the study may have been underpowered to detect small differences in relatively infrequent adverse events.

Although this study was conducted in a single high-volume Indian clinic, the findings have relevance for rabies-endemic countries worldwide, particularly in low- and middle-income settings where shortages of rabies immunoglobulin persist. Many such countries face the dual challenge of scaling up PEP access while managing constrained budgets and cold-chain infrastructure. Our demonstration that both single-agent and cocktail mAbs are safe and operationally feasible suggests that either product could be integrated into national PEP programmes, while recognising that local logistics, training, and supply-chain capacity may influence implementation.

The operational metrics assessed here, vials per patient and wastage are directly linked to programme efficiency and cost. Although Rabishield required slightly more vials per patient, wastage volumes were identical between the two products, suggesting that careful dose apportionment and trained infiltration techniques can offset potential losses from additional vial openings. Even without formal cost modelling, these data imply that the marginal increase in procurement cost for Rabishield may be modest, particularly when weighed against supply security and ease of integration. For ministries of health and global procurement agencies, such operational evidence is essential for optimising purchasing strategies, minimising stockouts, and ensuring that limited resources translate into maximal patient coverage.

## Conclusion

In conclusion, when used alongside vaccine for WHO category III exposures, both single-agent (Rabishield) and cocktail (Twinrab) rabies monoclonal antibodies were safe and operationally feasible in routine care. Wastage was identical between products, and the small excess in vial use with Rabishield did not translate into longer administration time, more injection sites, or reduced ease of infiltration. Programmes facing RIG shortfalls can adopt either product with confidence, allowing procurement terms, vial logistics, and local familiarity to guide choice; training in thorough wound infiltration and dose apportionment remains central to minimising wastage. Multi-centre pragmatic studies incorporating cost-effectiveness and supply-chain modelling, and stratified operational analyses (e.g., by age and wound site), are warranted to inform scale-up and policy.
